# Global workspace model of consciousness and its electromagnetic correlates

**DOI:** 10.4103/0972-2327.42933

**Published:** 2008

**Authors:** Ravi Prakash, Om Prakash, Shashi Prakash, Priyadarshi Abhishek, Sachin Gandotra

**Affiliations:** Central Institute of Psychiatry, Ranchi, India; 1 Department of Biotechnology, Delhi College of Engineering, Delhi, India; 2 Tata Consultancy Services, Pune, India; 3 Department of Clinical Psychology, Central Institute of Psychiatry, Ranchi, India

**Keywords:** Electromagnetic field, global workspace

## Abstract

The global workspace of consciousness was proposed in its elementary framework by Baars, in 1982. Since the time of inception, there have been many speculations and modifications of this theory, but the central theme has remained the same, which refers to the global availability of information in the brain. However, the present understanding about the origin of this global workspace or its mechanism of operation is still deficient. One of the less-studied candidates for this global workspace is the electromagnetic field of the brain. The present work is a brief review of the theoretical underpinnings of the Global workspace model, in terms of its theoretical framework and neuroimaging evidences. Subsequently, we turn towards another broad group of theories of consciousness, in the form of electromagnetic field theories. We then proceed to highlight some electromagnetic correlates derived from these theories for this global access phenomenon.

## Introduction

The past decade has achieved the landmark of demystifying the entity of consciousness, accomplished by the enduring efforts and well-thought out works of neuroscientists, mathematicians and philosophers. This can be seen as another evidence of correct scientific approach in the history of human efforts. Earlier evidences of this can be cited in the form of scientific explorations of gravitational force, origin of quantum mechanics and concepts of the origins of the universe.[1,2] In all these fields, nothing has been more spectacular than the convergence of expert brains from different disciplines, for the purpose of exploration of the unknown. This is most prominent in the field of consciousness studies, where physicists, biologists and mathematicians have established their individual roles. This is the reason for the explosion of theories in the field of consciousness research.

Of late, quite a number of interesting theories have been put forward from the discipline of cognitive sciences. Amongst these, one of the most popular theories in recent times has been the conscious access hypothesis, popularly known as Global workspace model of consciousness. The theoretical utilities, as well as the importance of this theory for explaining the various aspects of consciousness, have been proved by recent neuroimaging studies. However, the origin of this global workspace is a debatable issue. There have been various models to propose correlates to this global workspace model to ultimately reach the origin of consciousness. One of them is the electromagnetic field (EMF) of the brain, which has been inadequately studied in this context. The following is a brief review of the global workspace model of consciousness, including the theoretical underpinnings, evidences and applications. After reviewing the model of the global workspace, we proceed to the next step by proposing a correlate to this global workspace model through the electromagnetic field of the brain. We provide a model of this endogenous field and correlate its properties with the global workspace properties of consciousness. We refrain from stating that the global workspace model or the electromagnetic field of the brain can explain consciousness completely, but the spectacularity of the ability of the endogenous EMF to correlate with consciousness cannot be ignored and, in fact, should be a topic of further study.

## Consciousness as the Global Workspace

The theory of global workspace was first proposed by Baars BJ in 1982. However, the concept of global workspace is not so new. In 1959, John McCarthy made the first proposition suggesting the importance of global availability of information in the brain as a property of conscious events in his Pandemonium model.[3] Subsequently, Paul Rozin argued in favor of accessibility in the brain as the key factor in consciousness,[4] whereas Newell and Simon first showed the utility of a global workspace capacity in a complex system like the brain, without linking it to consciousness.[5]

As can be seen, different theorists have highlighted different aspects of the global workspace model. This is because the global workspace is multidimensional. Perceiving it through different dimensions yields different interpretations. This remains true even today, after about two and a half decades of the original proposition of the theory by Baars BJ. There have been various interpretations and ways of expressing the theory and conceptualising it.

Listing out all the viewpoints is out of the scope of this article. We, however, present two important perspectives, with a detailed interpretation of both, so as to give a broad idea about the basic conception of the theory. We will next turn to the study results, which strengthen the predictions of the theory. Subsequently, we will proceed to the implications of this theory, to explain the properties of consciousness.

### Global workspace from Baars' perspective

As Baars is the founder of the theory, we first analyse his perspective, which is predominantly cognitive.

As per his own account, ‘Consciousness is accomplished by a distributed society of specialists that is equipped with a working memory, called a global workspace, whose contents can be broadcast to the system as a whole.’[6]

Again he adds, ‘Global workspace theory suggests a fleeting memory capacity in which only one consistent content can be dominant at any given moment.’[7]

The theory encompasses the following two important themes:

1. *The brain is a distributed society of specialists:* Baars' connotation of these specialists is not as simple as it seems in the first instance. He does not mention any anatomical part of the brain as a representative of this specialist action in the brain. His specialist is not anatomical but computational. He points towards information processing events in the brain rather than to any neurophysiological or neuroanatomical substrate. According to him, the brain consists of many such specialists, which are continuously processing information [Figure 1].[6] His conceptualisation seems very similar to the Pandemonium model of McCarthy,[3] where he has proposed that the brain was a collection of demons, which were always doing something. The part of the activities which they do among themselves is the unconscious part, whereas their shouting at each other is the conscious part. He very intelligently expresses that there are events going on in the brain, which is very similar to the information-processing-specialists concept of Baars. These information processing events are hypothetical computational processes, similar to the symbol manipulation events originally proposed by Turing A.[8] The application of these computational processes in the neural machine grossly refers to a causal/mechanical process in which representational vehicles, for example language, are recognised and transformed in a semantically coherent fashion, purely on the basis of their syntactic properties. At a more subtle level, this means that the representational vehicles like language are broken up into the symbolic forms of the patterns of electrical transmission by neurons, which is based on the syntactic properties of the language. This data in the form of patterns of electrical impulses is then transformed in the brain into other patterns of electrical impulses, when it travels through innumerable synapses and neurons. These electrical activities (including action potentials and subthreshold potentials) are then recognised and manipulated by other neurons to produce output as needed by the computer.[9] Although the whole process of computation may seem abstract, it is the foundation of the highly sophisticated branch of Computational Neurosciences.

**Figure 1:**
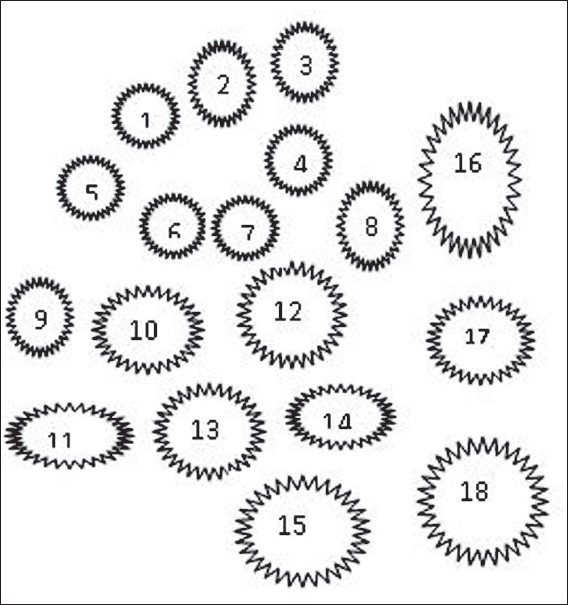
A schematic representation of various unconscious processes occurring in the brain. The different sizes have been made to signify different magnitudes and possible extent of neuronal involvement in these processes
2. *The society of the specialists is equipped with a global workspace:* This theme incorporates the most important part of the theory. According to this theory, the society of all the specialized computational processes in the brain is equipped with a unique memory. The exact meaning of the word memory is not clear here. In other words, there is no clear physical correlate of memory in this theory. This cannot be cited as the weak point of the theory because there is no clear meaning of Memory in the neuropsychology discipline as a whole. It becomes easier to understand Baars' meaning of memory, by going through the characteristics that he assigns to it:
  - *The memory is fleeting in nature:* As per the specifications provided by the proposer, there is no fixed capacity, location or constraints of time duration for this memory. It is fleeting in nature, which means that its composition as well as capacity change with time. Whether it becomes nil in a conscious state has not been specified, but theoretically it can become empty.
  - *The memory can only have one consistent content at a time:* The theory provides that the memory can have the content of only one information processing event at a time. It is unique for any memory function to have only one dominant content at a time; it is so for Baars' model of working memory.
  - *The content of the memory is decided by the consciousness:* The exact role of consciousness is to decide the dominant content of the memory. This decision is then executed by a top-down attentional amplification. This gives the entity of consciousness the power to choose one content to fill up the memory, from amongst the many information processing events in the brain. It is the content of this memory of which we become aware of or conscious of.
  - *The content of the memory can be broadcast to whole of the system*: The other important role of this working memory is that its content can be broadcast to the whole of the system. What Baars means is that once an information processing event becomes dominant in the working memory, the whole of the brain starts functioning to accomplish the information processing task of the memory content. At a more real level, the other parts of the brain computer (not necessarily the whole) start functioning in favor of the memory content. This will be observed as the involvement of distant and other unrelated brain areas, during the information processing event in the working memory. How this is performed will be dealt with in more details in the section where the evidences for this theory are discussed.

**Figure 2:**
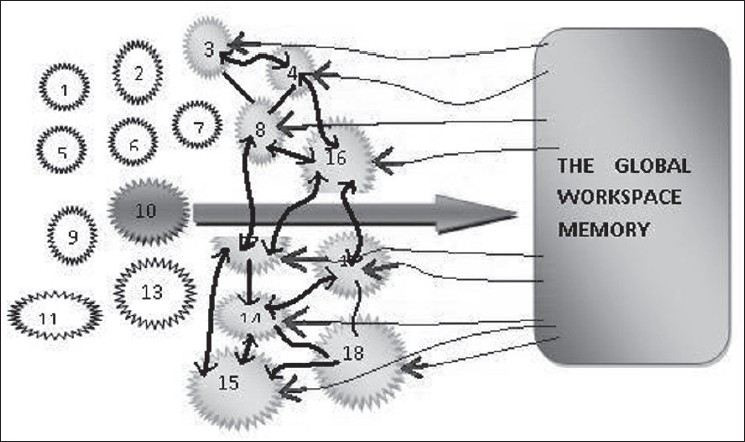
An example of functioning of the global workspace memory. An unconscious process (Circle number 10 for example) gains access to the global workspace by attentional amplification (the thick red arrow). The global workspace then propagates a widespread distribution (the thin red arrows) of the information to other information processing events in the brain (the light red circles). It also makes the information processing events connected to each other (the light black arrows), thus increasing the accessibility of information

As can be seen from the above, the working memory capacity operates in a unique way in which any information may be selected from the global brain scale and broadcasted and operated in a global scale [Figure 2]. For these reasons, this memory has also been termed the ‘Global workspace’ of the brain and the theory has been called the ‘Conscious access’ hypothesis, as the consciousness allows for wider access of the brain regions for this memory function.

### Global workspace from Dehaene's perspective

A short summary of the Dehaene and Nacchache's model of global neuronal workspace is as follows:

‘At any given time, many modular cerebral networks are active in parallel and processes information in an unconscious manner. Information becomes conscious, however, if the neural population that represents it is mobilized by top-down attentional amplification into a brain-scale state of coherent activity that involves many neurons distributed throughout the brain. The long distance connectivity of these “workplace neurons” can, when they are active for a minimal duration, make the information available to a variety of processes including perceptual categorization, long-term memorization, evaluation, and intentional action. We postulate that this global availability of information through the workplace is what we subjectively experience as a conscious state.’[10]

As can be seen from the above extract, Dehaene's description of global workspace is more specific, as compared to Baars' view. The following can be cited as the major specifications used in his model:

1. *Modularity of the cerebral networks:* Dehaene refers to cerebral networks similar to the specialists of Baars' model. These cerebral networks work as modules, which means that a group of networks sub-serves a fixed limited function. Further, according to this model, these networks are active in parallel, which means that they are active in their individual functions independent of the functions of other modules.
2. *Conscious and unconscious information:* This theory makes the explicit statement regarding the two types of information in the brain, which are: conscious and unconscious.
3. *Neural representation of information:* The most evident specification used in their model seems to be the fact that they explicitly link a neuronal population to one information in the brain. In addition, the model also mentions the mobilization of the neural population by top-down attentional amplification by the global workspace, so as to create a brain-scale state of coherent neural activity.
4. *The global workplace neurons:* Dehaene correlated the original global workspace with neurons, so much so that their model replaces this global workspace phrase with that of global workplace neurons. Again, there is no specific neuroanatomical location or designation of these Workplace neurons; it is a fleeting entity like that of the global workspace memory capacity in Baars' concept. In spite of its nonspecific location, Dehaene proceeds with the fact that the global workplace neurons show a long-distance connectivity. This long distance connectivity is, again, not a clear phenomenon, but his theory as well as his works subsequently highlight the importance of synchronicity in this connectivity phenomenon. It is this long-distance connectivity that he attributes to the phenomenon of global availability of information, leading to memorization, perceptual categorization and intentional action.

All in all, the theory proceeds one step ahead to incorporate neuronal activities in the global workspace, so that neural phenomenon, like synchronicity, is a signature of the global availability of information, which mediates the long distance connectivity of the brain regions termed as modular units.

### Other perspectives of the global workspace

Recently, there have been a number of speculations and applications of the global workspace model. Prominent among the many models are those of Crick[11] and Koch and Edelman.[12] Maia and Cleeremans have recently proposed a model called the Connectionist Model, where they have proposed that the global workspace is actually a level of coalition/connectivity of neurons, which can be correlated by the strength and sustainability of neuronal firing. Strong and sustained firing increases the likelihood of the corresponding representation being a part of the winning neuronal coalition. Conversely, neurons that are a part of a winning coalition receive excitation from other neurons in the coalition, which results in an increased firing rate.[13]

## Theoretical and experimental evidences of the global workspace

Ever since the theory was proposed in 1982, there has been an increasing accumulation of evidence in favor of the existence of global workspace, either as a physical or functional entity in brain. A few examples of supporting evidences are given below:

### Conscious perception

It is a known fact that several of the sensory analytic processes in the brain are unconscious. This gave rise to an important question: what is the difference between conscious and unconscious perception?. A satisfactory answer was provided by the global workspace model, where it was shown that more conscious perception involves more of the brain regions. Dehaene *et al.* have recently shown that backward-masked visual words mainly activate the visual cortex, whereas identical conscious words evoke widespread visual, parietal and frontal activation. Similarly, Tononi *et al.* and Srinivasan *et al.* have demonstrated in binocular rivalry that conscious visual input invokes more intense and coherent Magneto encephalogram (MEG) responses from flicker-tagged input than a matched unconscious stream.[14,15]

### Conceptualising working memory

Working memory (WM) is a very important concept in cognitive neurosciences. In spite of the fact that its active components of perceptual input, rehearsal and recall are conscious elements, the origin of WM and its neural mechanisms are poorly understood. The model of global workspace was the first to give WM a distinct and detailed framework,[16] which can be said to be the most accepted model in cognitive neurosciences. It underscored an important property of WM, which was its ability to perform widespread recruitment of neurons with the help of attentional involvement. Consistent with this theoretical predication,

John *et al.* found that quantitative EEG across six WM tasks showed the involvement of widespread cortical components, accounting for 90% of the variance.

### The controversy of voluntary control

The controversy of voluntary control of an action is far from resolved. However, several evidences suggest that similar complex tasks can happen under voluntary control or involuntarily.[17,18] In spite of the controversial status of volition, it has been used as an experimental variable in several experiments that have yielded interesting evidences in favor of the global workspace theory. Conscious feedback training experiments have provided spectacular examples of the scope of access to almost any neuronal populations and even single spinal motor neurons.[19,20] This is consistent with the conscious access theory, which postulated that consciousness allows for wider recruitment of neurons.

### Unconscious versus conscious input processing

In contrast to the conscious processing which involves widespread brain areas, unconscious processing has been shown to be quite limited. This has been shown by the failure to demonstrate multiple-word subliminal priming in spite of repeated efforts,[21] as well as failure of elicitation of multiple-word effects (a typical example of conscious processing) in unattended listening.[22] It thus seems that consciousness is required for integration of various brain regions and, therefore, wider involvement too.

## Possible mechanisms of the global access

The mechanisms of the global access in the brain are not known at present. However, several neurological correlates have been proposed by different investigators:

1. Complex re-enterant thalamocortical dynamics[23,24]
2. Gamma synchrony[25]
3. NMDA synapses[26]
4. Thalamic hub[27]

The above-mentioned neuroanatomical correlates are a few of the many and the list is exhaustive.

## Electromagnetic field theories of consciousness

Needless to say, consciousness has been viewed from different frames of references, which has resulted in the production of different theories. But a closer look at these various theories reveals the fact that there are many similarities among them. It will be more yielding if these theories are used in connection with each other for covering the wider aspects of consciousness. We now turn towards a group of theories, which have focussed on the electromagnetic field of the brain and find out that how these theories produce an important correlate of the global workspace in the form of the electromagnetic field of the brain.

There are two main theories at present, which are: Conscious electromagnetic field theory (CEMI) of John Joe McFadden and the Electromagnetic theory of consciousness by Susan Pockett. We do not intend to describe these theories at length. Instead, we will point out the possible electromagnetic correlates yielded from these and other electromagnetic field theories for the global workspace mentioned above, after a brief description of the basics of generation of electromagnetic field in the brain.

## Generation of electromagnetic field in the brain

**Figure 3:**
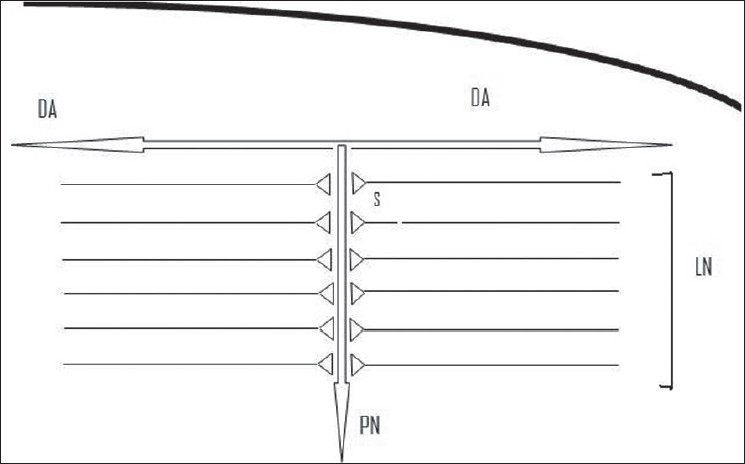
A schematic diagram of a typical pyramidal neuron (PN) in the neocortex along with its dendritic arborisations (DA). The laminar organisation of the neurons (LN), which synapse (s) on these neurons (the triangular knobs), result in the amplification of the local field potential generated by these neurons

As is well-known, the endogenous electromagnetic field of the brain is a highly structured entity. There are two main sources contributing to the formation of this field. These are the fields produced from the neuron firing and the fields generated by the movement of ions into and out of cells and within extracellular spaces. The electrical field at any point in the brain will be a superposition of the induced fields from all the neurons in the vicinity (superimposed on the fields generated by ion movement) and will depend on their firing frequency, geometry and dielectric properties of the tissue. For neurons that are arranged randomly, their induced fields will tend to sum to zero. However, there are several brain structures, where the organisation of neurons is laminar [Figure 3]. These structures include the neocortex, hippocampus etc. This laminar organisation of neurons tends to orient neurons along the direction of the field produced by other parallel neurons, thereby amplifying the local field potential. Consequently, endogenous electromagnetic (em) fields of tens of volts per meter at the level of neurons are capable of generating fields of several tens of thousands of volts per meter, translating to up to several millivolts across the 5 nm neuronal cell membrane.[28,29]

In more biophysical terms, the ionic currents in the brain cause electrical and magnetic fields obeying Maxwell's and Ohm's laws. These fields have direction and magnitude. The value of the electrical potential at a point $\vec{{\text{r}}_{\text{0}}}$ in a volume conductor from injected current densities $\vec{{\text{J}}_{\text{i}}}$ (suppose from a group of synapses in a small cube of pyramidal neurons in above figure) injected at points $\vec{\text{r}}$ which are distances R from ${\text{r}}_{0}(\vec{\text{R}}=|\vec{\text{r}}-\vec{{\text{r}}_{0}}|)$ is given by the Poissons equation:

$$\text{V}(\vec{\text{r}}-\vec{{\text{r}}_{0)}}=-1/4\text{π}\int_{\text{vol}}(\text{div}\vec{{\text{J}}_{\text{i}}}/\text{R}){\text{d}}^{3}\text{r}$$

Where Div J is the divergence of the vector J, which is to be understood as the differentiation of the vector which it presents.[30]

## Electromagnetic field of the brain for the global workspace: Candidate or correlate

### Electromagnetic field influence on neurons: McFadden's Candidate viewpoint

As is evident from the above section, much has been explored about the effects that neuronal events have on the endogenous electromagnetic field of the brain. But much less explored is the fact that this endogenous EMF can influence the neuronal events.

Through the CEMI field theory, McFadden showed that this information rich EMF modulates the neuronal firing through electrical coupling. Electrical coupling can occur in two ways: by modulation of the amplitude of field disturbances or by shifting neuronal transmembrane potential to adjust electrical excitability. The second event is more probable, because it is a less energy consuming phenomenon. Thus, the final outcome of the electrical coupling is generally the adjustment of the electrical excitability of the neuron. This adjustment can in turn inhibit or facilitate neuronal firing by modulating the drop in membrane potential from the existing level to the threshold needed for firing (-40mV in the case of neurons). In the second condition, the excitability of the receiver neuron will depend on many factors, the most important being the closeness of the existing membrane potential to the firing potential.[28]

**Figure 4:**
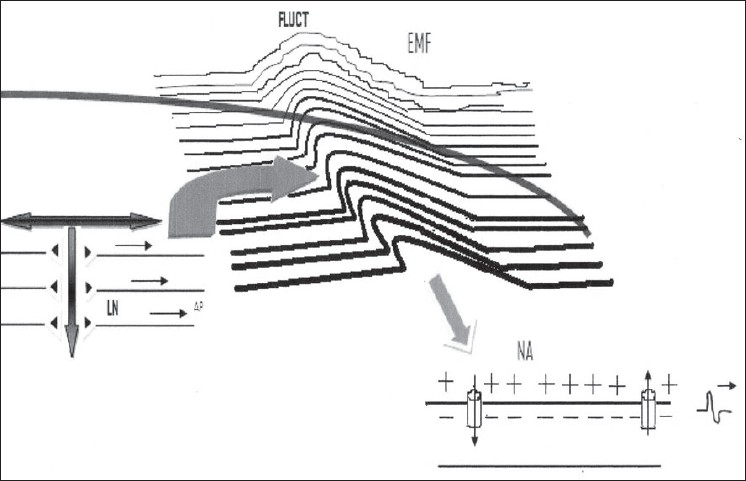
A schematic representation of the CEMI field theory of consciousness. The synchrony of action potential (AP) and the laminar organisation of neurons (LN) in the neocortex allow for the phase-locking of the resultant local electromagnetic field fluctuation (FLUCT), thus increasing the intensity of the field by several folds. This fluctuation in turn can have effects on the near and distant electromagnetic properties of neurons, represented here by one neuronal axon (NA). This impact can be theoretically so intense that it can result in generation of action potential in the neuron

The other important factor is the orientation of the neuron with respect to the em field, with the maximum sensitivity being for the neurons which are bent relative to the isopotential lines of the em field. In this way, firing of a neuron can influence the firing of a distantly located neuron through the em field. This impact is further increased when a large number of neurons fire in a synchronous manner, as synchronicity amplifies the em field effects by phase-locking em field fluctuations generated by distributed neurons. The effect of the field coupling generated by this synchronous firing can be (theoretically) so intense that it can fire a group of neurons, which can then be translated into motor or cognitive task [Figure 4]. As per the hypothesis of the CEMI field theory, consciousness is the component of the brain's electromagnetic information field that is transmitted to motor neurons and is thereby capable of communicating this state to the outside world.[31]

A closer look at this theory provides us with a candidate for the conscious access hypothesis. According to the global workspace hypothesis, consciousness allows global access of the brain. In terms of the CEMI field theory, this global access is actually due to synchronization of firing of cortical neurons by the mechanism of phase-locking. In conscious visual perception, for example, many parietal and frontal areas have been observed to be involved, in addition to primary visual area, in accordance with the global workspace hypothesis.[10,32] At the same time, studies using multiple electrodes implanted in the brain of experimental animals have demonstrated that the cluster of neurons in their visual cortex fire in synchrony, when they perceive visual stimuli.[33] Thus it seems that this synchrony correlates with the recruitment of wider cortical area during conscious perception. There are also indirect evidences that synchronous firing also correlates with awareness and attention in man. The reverse of this phenomenon has also been found. Anaesthetic state, a clearly known state of almost no cortical involvement, has also been found to be associated with the loss of gamma band activity and loss of coherence across major quadrants of the cortex.[34]

Studies have also found that during the performance of tasks that require great mental effort, more of the frontal cortical areas are involved, in addition to the areas involved in simple tasks.[35] On the other hand, fractal dimension of EEG signal during various cognitive states has shown that the dynamic complexity is increased during creative thinking, as compared to deep sleep.[36] Thus it seems that the complexity in conscious thinking, involving a wider brain area at the same time, also correlates with an increase in the complexity of the brain's em field.

Thus theoretical as well as experimental evidences suggest that the brain's electromagnetic field may be a potential candidate for global workspace and thereby a substrate for consciousness.

### Conscious electromagnetic patterns: Pockett's Correlate viewpoint

**Figure 5A and B:**
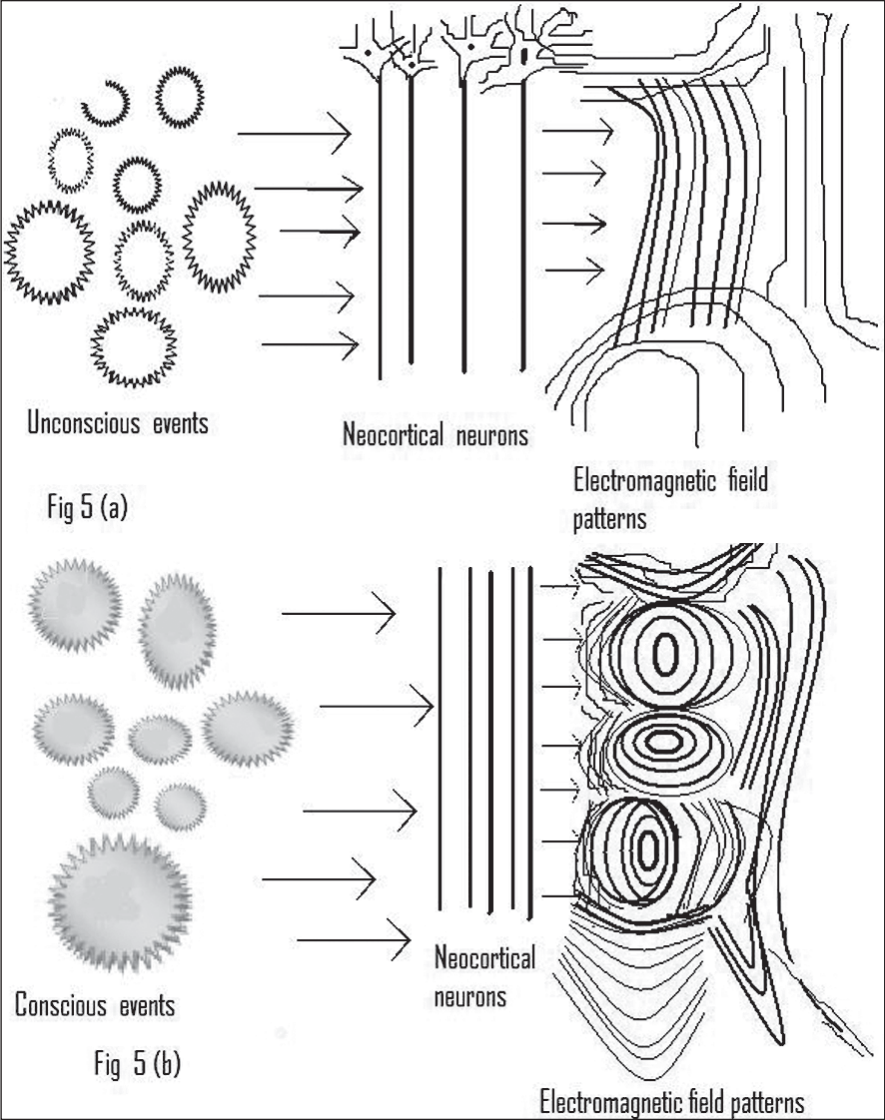
Pockett's Correlate model of consciousness. The electromagnetic field of the brain during unconscious events (represented by stars in Fig. 5A) are less complicated and less dynamic, as compared to the electromagnetic field configurations during conscious events. These electromagnetic field patterns are unique to the human brain. Thus, consciousness is only indirectly related to the electromagnetic field patterns of brain

Soon after the proposal of the CEMI theory by McFadden, Susan Pockett raised serious objections on any such concept which proposed that the electromagnetic field of the brain could have an influence on neuronal firing. According to her, the electromagnetic properties of the brain cannot be explanatory to give rise to consciousness. However, she did agree with its correlatory properties with conscious states. As per her arguments, the human brain is the only object known till date, that has unique spatiotemporal patterns, which result from specific conscious states.[37] These states, as she explains, are the correlates of consciousness rather than consciousness itself [Figure 5]. The backdrop of this concept, in her own account, has been the works of Freeman, where he recorded the electromagnetic field from the surface of the brain of rabbits, in two different states: the state of experiencing an odor (the conscious state) and the state where there is no such similar experience (the unconscious state). The electromagnetic fields in these states were the same, in terms of frequency, amplitude and phase properties. However, striking differences were observed in the spatial patterns of these states.[38–40] At present, we do not have enough sophisticated equipments, by which we can measure these patterns. We also do not have a solution to the inverse problem, so as to correlate with the spatiotemporal patterns of the human EEG. As per this theory, we cannot step further in terms of electromagnetic correlates of consciousness, until we qualify on the above mentioned criteria.[37]

However, this theory coincides with the global CEMI field theory on one point, which is interestingly, our point of interest also. When we take a closer look at Freeman's experiments, we see that the spatiotemporal patterns of the conscious state in a rabbit are more complex, as compared with the unconscious state. It is this complexity, in fact, which refrained Pockett from further analysis, keeping in view the lack of sophisticated equipments. A similar increase in the dynamic complexity of EEG has been cited in the CEMI field theory of McFadden in states of creative thinking, as compared to deep sleep.[36] This increase in dynamic EEG complexity is, in fact, a strong indicator of the involvement of more neurons across the brain. This is, again, consistent with the global workspace model, which postulated the globalization of information in conscious states.

## Conclusion

The global workspace model of consciousness has received consensus in the past two decades. Although different perspectives have yielded different frameworks, the core of the theory remains a global availability of information in the brain, provided by attentional amplification. The well-explained functional aspect of the model is evidently contrasted by poorly understood origin of this global workspace. In the list of some hypothetical candidates for this model, the electromagnetic field of the brain holds a special position, given its ability to explain some important aspects of this global access phenomenon.

The theory remains controversial due to our inadequate understanding of the spatiotemporal patterns of the electromagnetic field of the brain. However, the theory is worth exploration and studying with regard to the idea that mechanisms of global access of information of the brain will definitely yield more correlates of the global workspace.
